# Multispecies comparisons of adaptability to climate change: A role for life‐history characteristics?

**DOI:** 10.1002/ece3.3517

**Published:** 2017-11-01

**Authors:** Sarah T. Saalfeld, Richard B. Lanctot

**Affiliations:** ^1^ Migratory Bird Management Division US Fish and Wildlife Service Anchorage AK USA

**Keywords:** arctic, phenological advancement, phenological mismatch, phenotypic plasticity, shorebirds, trophic mismatch

## Abstract

Phenological advancement allows individuals to adapt to climate change by timing life‐history events to the availability of key resources so that individual fitness is maximized. However, different trophic levels may respond to changes in their environment at different rates, potentially leading to a phenological mismatch. This may be especially apparent in the highly seasonal arctic environment that is experiencing the effects of climate change more so than any other region. During a 14‐year study near Utqiaġvik (formerly Barrow), Alaska, we estimated phenological advancement in egg laying in relation to snowmelt for eight arctic‐breeding shorebirds and investigated potential linkages to species‐specific life‐history characteristics. We found that snowmelt advanced 0.8 days/year—six times faster than the prior 60‐year period. During this same time, six of the eight species exhibited phenological advancement in laying dates (varying among species from 0.1 to 0.9 days earlier per year), although no species appeared capable of keeping pace with advancing snowmelt. Phenological changes were likely the result of high phenotypic plasticity, as all species investigated in this study showed high interannual variability in lay dates. Commonality among species with similar response rates to timing of snowmelt suggests that nesting later and having an opportunistic settlement strategy may increase the adaptability of some species to changing climate conditions. Other life‐history characteristics, such as migration strategy, previous site experience, and mate fidelity did not influence the ability of individuals to advance laying dates. As a failure to advance egg laying is likely to result in greater phenological mismatch, our study provides an initial assessment of the relative risk of species to long‐term climatic changes.

## 1 INTRODUCTION

Phenological advancement in response to climate change has been well documented in a wide variety of taxa (Crick, Dudley, Glue, & Thomson, 1997; Forchhammer, Post, & Stenseth, 1998; Parmesan & Yohe, 2003; Post, Forchhammer, Stenseth, & Callaghan, 2001; Stenseth et al., 2002; Walther et al., 2002). By advancing phenology, individuals can time life‐history events to the availability of key resources so that individual fitness is maximized. In many species, optimal breeding occurs so that peak food demands (e.g., developing young) coincide with peak prey availability (Bronson, 1985; Durant, Hjermann, Ottersen, & Stenseth, 2007; Visser, Holleman, & Gienapp, 2006). However, as optimal breeding periods can vary based on annual environmental fluctuations, individuals may adjust their annual phenology through phenotypic plasticity with individuals responding to environmental conditions in the same year, via learning, or via maternal effects on their offspring (Visser, 2008). The ability of individuals to adjust phenology, however, may depend on several intrinsic factors such as experience (Whelan, Strickland, Morand‐Ferron, & Norris, 2016), life‐history characteristics (Kerby & Post, 2013), or trophic level (Thackeray et al., 2016). Such variable responses among species could result in a phenological mismatch between the timing of food requirements and the availability of prey (Both, van Asch, Bijlsma, van den Burg, & Visser, 2009; Brook, Leafloor, Abraham, & Douglas, 2015; Doiron, Gauthier, & Lévesque, 2015; Durant et al., 2007; Gaston, Gilchrist, Mallory, & Smith, 2009; Harrington, Woiwod, & Sparks, 1999; Visser, van Noordwijk, Tinbergen, & Lessells, 1998; Visser et al., 2006), ultimately leading to long‐term population declines (Both, Bouwhuis, Lessells, & Visser, 2006; Both et al., 2010; Møller, Rubolini, & Lehikoinen, 2008; Saino et al., 2010).

Phenological mismatch may be especially apparent in the highly seasonal arctic environment, which is experiencing the effects of climate change more so than any other region, resulting in earlier, warmer, and longer summers (Callaghan et al., 2005; Hodgkins, 2014; Serreze & Francis, 2006). Shorebirds comprise a large portion of the avian fauna breeding in the Arctic and are an ideal taxa to investigate phenological mismatch, as they time their long‐distance migrations using a combination of endogenous and photoperiod cues (Karagicheva et al., 2016; Piersma, Brugge, Spaans, & Battley, 2008), but rely on a short pulse of abundant food whose emergence is dictated by local climatic conditions on the breeding grounds (Bolduc et al., 2013; Danks, 1999; Tulp & Schekkerman, 2008). Upon arrival, shorebirds use the presence of snow to time egg laying, as egg laying is inhibited until enough snow‐free land is present (Grabowski, Doyle, Reid, Mossop, & Talarico, 2013; Green, Greenwood, & Lloyd, 1977; Liebezeit, Gurney, Budde, Zack, & Ward, 2014; Meltofte, 1985; Meltofte, Høye, Schmidt, & Forchhammer, 2007; Smith, Gilchrist, Forbes, Martin, & Allard, 2010). By doing so, shorebirds may be able to accurately time hatch with peak food availability, as timing of snowmelt, along with temperatures after snowmelt, influences insect emergence (Bolduc et al., 2013; Danks, 1999; Høye & Forchhammer, 2008; Tulp & Schekkerman, 2008). However, with earlier and warmer summers, decoupling of this synchronization may occur if (1) shorebirds fail to advance arrival dates, arriving after snow has already melted, or (2) the time between snowmelt and insect emergence shortens (due to warmer temperatures), but shorebird incubation periods remain the same (Saalfeld et al. unpublished data). Such a decoupling has the potential to greatly impact shorebird populations, as prey availability directly influences juvenile growth and survival rates (McKinnon, Nol, & Juillet, 2013; McKinnon, Picotin, Bolduc, Juillet, & Bêty, 2012; Pearce‐Higgins & Yalden, 2004; Reneerkens et al., 2016; Schekkerman, Tulp, Piersma, & Visser, 2003; Senner, Stager, & Sandercock, 2016). Therefore, understanding the ability of species, as well as the life‐history characteristics that enable a species to adapt to environmental change, is crucial for determining long‐term population viability in the face of a changing climate. However, few studies have investigated phenological advancement in laying dates in multiple shorebird species at the same site over a long period of time, allowing for direct comparisons among species under the same environmental conditions (Pearce‐Higgins, Yalden, & Whittingham, 2005; Gill et al., 2014; but see Høye, Post, Meltofte, Schmidt, & Forchhammer, 2007; McKinnon et al., 2012; Grabowski et al., 2013; Liebezeit et al., 2014). Thus, our objectives for this study were to determine (1) the level of phenotypic plasticity in nest initiation dates in relation to snowmelt in eight arctic‐breeding shorebird species nesting over a 14‐year period, and (2) how a species’ migration pattern, timing of nesting, and settlement strategy (and by association an individual's mate and site experience) relate to their ability to adjust egg laying in response to earlier onset of summer.

We predicted that species with long‐distance migrations would be less able to advance egg laying in response to earlier onset of summer, as these species use migration cues that are unrelated to climatic conditions on their breeding grounds (Both et al., 2010; Doxa et al., 2012; Jonzén et al., 2006; Saino et al., 2010; Visser, Both, & Lambrechts, 2004). Additionally, the rigid schedules and the sheer distances long‐distance migrants travel may further constrain phenological advancement in these species (Helm, Gwinner, & Trost, 2005). In contrast, we predicted that short‐distance migrants would be better able to accurately time their nesting phenology, as their wintering site conditions are likely more indicative of conditions on their breeding grounds (Both et al., 2010; Doxa et al., 2012; Jonzén et al., 2006; Saino et al., 2010; Visser et al., 2004). In addition to distance, other factors such as location of wintering site, northbound migration route, and whether a species travels over land or water could also influence the ability of species to adjust egg laying, as these factors may influence the reliability of species to predict conditions on the breeding grounds during migration. In addition to changes in arrival dates, species may also be able to advance egg laying by reducing the time between arrival and egg laying (Visser et al., 2004). However, as early‐nesting species may already be nesting as early as possible, we predicted that late‐nesting species would be better able to adjust egg laying in response to earlier onset of summer, assuming the period between arrival and breeding is longer than in early‐nesting species. Finally, we were unable to predict whether opportunistic or conservative species (Saalfeld & Lanctot, 2015) would be better able to adjust egg laying, as both species exhibit traits that may enhance their ability to adjust to annual conditions. For example, opportunistic species may benefit by selecting favorable breeding locations on an annual basis, allowing them to adjust to annual climatic conditions (Kempenaers & Valcu, 2017; Lanctot & Weatherhead, 1997; Lanctot et al., 2016), while conservative species (that exhibit high site fidelity) may benefit by learning from the past experiences (Visser, 2008) or repairing with prior mates that would allow for earlier egg laying (Soikkeli, 1967; Jehl, 1973; Miller, 1983; Jönsson, 1987; but see Sandercock, Lank, & Cooke, 1999).

## 2 MATERIALS AND METHODS

### 2.1 Study area

**Figure 1:**
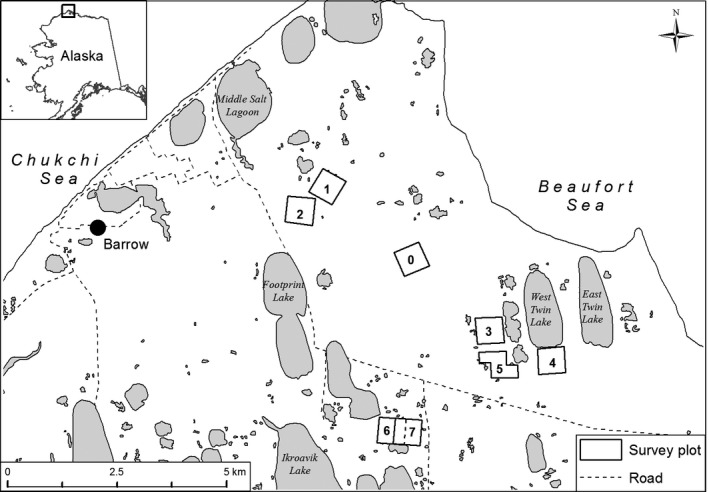
Location of shorebird study plots near Utqiaġvik (formerly Barrow), Alaska, 2003–2016. Dashed line in plot 7 illustrates eastern plot edge of plot 6 before it was moved to the east to create plot 7

From 2003 to 2016, shorebird nesting and snowmelt data were collected on eight 36‐ha plots near Utqiaġvik (formerly Barrow), Alaska (Figure 1). Plots were selected based on previous research activities in the area, accessibility from the road system, and suitable densities of shorebird nests. The eight plots reflected the general diversity of habitat types and species of shorebirds found nesting in the Utqiaġvik vicinity. Plots 1–3 were monitored in all 14 years; plots 4 and 6 were monitored only in 2003 and 2004, respectively; plots 5 and 7 were monitored from 2004 to 2016 (13 years) and 2005 to 2016 (12 years), respectively; and plot 0 was monitored from 2013 to 2016. We divided all plots into 144 quadrants (50 × 50 m) using wooden stakes placed every 50 m to facilitate data collection. Habitat within the study plots consisted mainly of tundra dominated by sedges, grasses, and moss interspersed with small ponds. This created a mosaic of low, wet marsh habitat and higher, well‐drained upland habitat (Brown, Everett, Webber, MacLean, & Murray, 1980).

### 2.2 Data collection

#### 2.2.1 Nest initiation dates

Shorebird nests were located using single‐person area searches, two‐person rope drags, and opportunistically during most days between early June and early July (see Saalfeld & Lanctot, 2015 for description of methods and effort). We visited nests found with less than four eggs (modal clutch size for all species) daily until clutches were completed or until clutch size remained unchanged for two consecutive days. For clutches found during incubation, we floated 2–4 eggs to determine incubation stage (accuracy of which was typically within 4 days of the true hatch date; Liebezeit et al., 2007). We estimated nest initiation dates (i.e., date first egg laid) for nests found during laying (14% of nests) by assuming one egg was laid per day, and for nests found during incubation by (1) subtracting a species’ incubation period (based on the Birds of North America accounts; Poole, 2005) from hatching dates for successful nests (59% of nests) or (2) using estimated development age based on flotation for unsuccessful nests (27% of nests). Our ability to find and estimate nest initiation dates for the majority of nests was likely enhanced by high nest survival rates in this region (Lanctot et al. unpublished data) due to an active fox removal program that was initiated in 2005 (Barto, Pratt, & Sinnett, 2015).

#### 2.2.2 Adult shorebird capture, resighting, and mate and site fidelity

We captured adult shorebirds on nests using a modified luchock trap (or bow‐net; Priklonsky, 1960) and marked them with a US Geological Survey metal leg band, a unique combination of colored leg bands and a single dark green flag, uniquely engraved for a few species. We documented the return of individuals by capturing them or resighting them at nests, the latter usually at distances <3 m from observers where bands could easily be seen. Our extensive nest searching and capture/resighting effort allowed us to document the return of individuals among years and whether individuals mated with a new or the same individual.

#### 2.2.3 Timing of snowmelt

We estimated the percentage of snow cover within 36, 50 × 50 m quadrants equally spaced throughout each study plot to the nearest 5% every 2–5 days until ≤10% snow cover remained. We estimated the mean snow cover across all quadrants on each plot on a given date and then linearly regressed these values through time to determine the date when 20% snow cover was present on each plot in each year. We chose 20% as our cutoff value, as shorebirds begin initiating nests once enough snow‐free land is available (Grabowski et al., 2013; Green et al., 1977; Liebezeit et al., 2014; Meltofte, 1985; Meltofte et al., 2007; Smith et al., 2010). While several studies have used 50% as their cutoff value (Smith et al., 2010; Grabowski et al., 2013; but see Liebezeit et al., 2014), we chose 20%, as it could be calculated in most years. As the annual date for 20% snow cover was highly correlated (*r *=* *.91, *n *=* *11) with the date of 50% snow cover for years when data were available, this particular cutoff value likely had little impact on our results. In both 2009 and 2016, snow cover was present, but <20% on one plot during the first snow survey. Therefore, because winter winds keep snow from accumulating on the tundra and snow melts rapidly once temperatures reach 0°C, we used the date prior to the first survey as a conservative estimate of 20% snow cover for these plots. We also excluded nests on two plots lacking snowmelt information in 2004 when relating nest initiation dates to date of 20% snow cover.

### 2.3 Data analyses

#### 2.3.1 Phenology of snowmelt

To control for spatial variation in snow accumulation and melt, we used snowmelt measurements recorded at the plot level within each year for all analyses. We assessed the level of annual variability in timing of snowmelt by documenting changes in the mean date of 20% snow cover within plots among years. Additionally, we determined if timing of snowmelt advanced in the past 14 years by relating the date of 20% snow cover within plots to year using a general linear mixed model with plot as a random effect (PROC MIXED with standard variance components; SAS 9.4, SAS Institute, Cary, NC).

#### 2.3.2 Phenology of egg laying

To determine the ability of shorebirds to adjust their timing of egg laying in response to variable snowmelt, we first determined if nest initiation dates were influenced by timing of snowmelt by relating individual nest initiation dates to date of 20% snow cover using a general linear mixed model with plot as a random effect. Next, we determined whether shorebirds have advanced their laying dates over the 14‐year study by relating nest initiation dates to year using a general linear mixed model with plot as a random effect. These and all subsequent analyses were conducted separately for each species after removing years with inadequate sample sizes (i.e., <5 nests per year).

Because renesting can potentially bias our understanding of relationships between snowmelt and nest initiation, we removed all known renests occupied by individuals who had been identified with an earlier nest in the same season. Additionally, we repeated analyses after removing the last 25% of nests laid to assess the effect of including potential renests. We chose this approach because renests typically occur later in the season, but are difficult to document (Gates, Lanctot, & Powell, 2013; Naves, Lanctot, Taylor, & Coutsoubos, 2008). However, as similar estimates and significance were obtained after removing the later‐laid nests, we present results with all data included.

#### 2.3.3 Role of migration pattern, timing of nesting, and settlement strategy on timing of egg laying

We determined whether wintering location, migration pattern (distance and migration route), timing of nesting (mean nest initiation dates), or settlement strategy (conservative or opportunistic) was related to a species’ ability to respond to changing snowmelt conditions by qualitatively comparing the slope parameter from the regression of nest initiation date and date of 20% snowmelt among species with differing life‐history characteristics (see Table 1). We chose to use this relationship, instead of the relationship with year, as not all species had sufficient data in all years, potentially biasing comparisons. All species, however, were observed for the entire range of snowmelt conditions (e.g., data were available from both early and late snowmelt years).

**Table 1:** Life‐history characteristics (i.e., wintering locations, migration distances and routes, mean nest initiation dates, and settlement strategies) of shorebird species nesting near Utqiaġvik (formerly Barrow), Alaska from 2003 to 2016

Species^a^	Wintering location and migration distance^b^	Northbound migration route^b^	Mean nest initiation date^c^	Settlement strategy^d^
Dunlin	Southeast and East Asia (M)	East Asian and East Russian coasts (L/W)	11 June	Conservative
Semipalmated Sandpiper	Northern and Central coasts of S. America (M)	Interior N. America (L)	11 June	Conservative
American Golden‐Plover	Southern S. America (L)	Interior N. America (L)	16 June	Both
Long‐billed Dowitcher	Southern N. America (S)	Pacific coast and interior of N. America (L)	21 June	Both
Red‐necked Phalarope	Pelagic off west coast of S. America (L)	Offshore and along Pacific coast (L/W)	18 June	Both
Red Phalarope	Pelagic off west coast of S. America (L)	Offshore along Pacific Coast (W)	14 June	Opportunistic
Pectoral Sandpiper	Southern S. America (L)	Interior N. America (L)	16 June	Opportunistic
Western Sandpiper	Pacific coast from California to Peru (M)	Pacific coast of N. America (L)	16 June	Both

a Species ordered based on the slope of regression line between nest initiation date and date of 20% snowmelt (see Figure 3).

b Wintering location, migration distance (L = long, M = medium, S = short), and northbound migration route (L = land, W = water) derived from Birds of North America (Poole, 2005).

c Mean nest initiation date calculated from 2003 to 2016.

d Settlement strategy derived from Saalfeld and Lanctot (2015). See text for traits of conservative and opportunistic species. “Both” is used when species have both conservative and opportunistic traits.

#### 2.3.4 Individual phenotypic plasticity in timing of egg laying

For species that regularly returned to the study area to breed in successive years, we determined whether an individual tended to always nest at the same time within a calendar year by calculating the number of days between nesting attempts in different years. Next, we investigated how the timing of egg laying varied among individuals compared to within individuals for each species. To do this, we calculated the repeatability of laying dates between years for individuals following Lessells and Boag (1987), in which variance components were calculated from the mean squares from an analysis of variance (PROC ANOVA; SAS 9.4, SAS Institute, Cary, NC). Repeatability (*r*) ranges from −1 to +1, where positive values represent greater variation among individuals and negative values represent greater variation within individuals. These analyses were done for three species that each had ≥48 individuals observed for 2–9 years: Dunlin (*Calidris alpina*), Semipalmated Sandpiper (*Calidris pusilla*), and Red Phalarope (*Phalaropus fulicarius*).

As phenotypic plasticity may vary between the sexes, we repeated analyses for each sex separately. Additionally, we repeated analyses using only one individual from a given nest to determine whether including both individuals from a nest biased results. Because similar estimates and significance were obtained for these restricted datasets, we present results with all data included.

#### 2.3.5 Role of experience and mate fidelity on timing of egg laying

To determine if individuals learned from past nesting experiences, we investigated whether individuals adjusted their date of nest initiation relative to when they nested in their first year. To do this, we related the difference between laying date and date of 20% snow cover in the initial capture year to the difference in the following year using a general linear model (PROC GLM; SAS 9.4, SAS Institute, Cary, NC). We expected individuals that nested too early (e.g., before snow had melted) in their initial year to adjust their laying dates to be later in their second year and vice versa. These analyses were restricted to the three species (i.e., Dunlin, Semipalmated Sandpiper, and Red Phalarope) with adequate return rates (see above).

Next, we determined whether mate fidelity affected an individual's date of egg laying by comparing differences between nest initiation dates and date of 20% snow cover for individuals that nested with the same mate versus individuals that nested with a new mate using an analysis of variance. Here, we restricted analyses to individuals observed in both their initial capture year and the year after and for which their mate was identified in both years. We categorized nests in the following year as either exhibiting mate fidelity (i.e., nests with both members of the original pair) or not (i.e., nests with one member of the original pair, but a new mate). These analyses were restricted to biparental incubators with adequate return rates (i.e., Dunlin and Semipalmated Sandpiper). Given low sample sizes and the insignificance of sex above, we combined information for both sexes in these analyses.

## 3 RESULTS

### 3.1 Phenology of snowmelt

**Figure 2:**
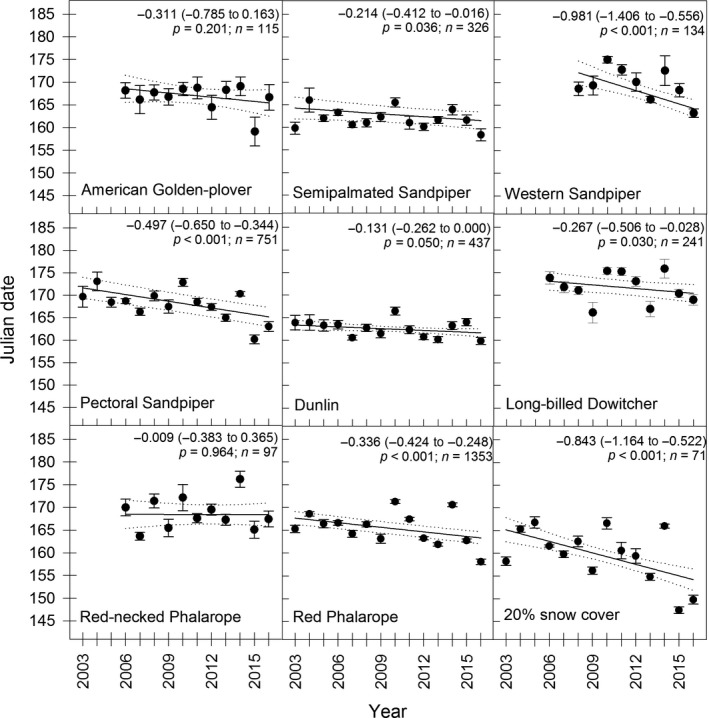
Observed (mean ± *SE*) and model predictions (line plot with 95% confidence intervals) for the advancement of snowmelt (bottom right graph) and shorebird nest initiation dates (other graphs) from 2003 to 2016 near Utqiaġvik (formerly Barrow), Alaska. Julian date 145 = 25 May (24 May in leap years). Parameter estimates (±*SE*) and *p*‐values for the effect of year, along with sample sizes are presented at the top of each panel for each species and 20% snow cover

During the past 14 years, we observed large annual variability in timing of snowmelt, with the mean date of 20% snow cover ranging from the 28 May to 16 June (Figure 2). Despite this large interannual variability, we detected a significant negative relationship between year and date of 20% snow cover (*F*_1,63_ = 26.44; *p *<* *.001), with models predicting the date of 20% snow cover occurring 11 days earlier in 2016 as compared to 2003 (advancement rate of 0.8 days/year; Figure 2).

### 3.2 Phenology of egg laying

**Figure 3:**
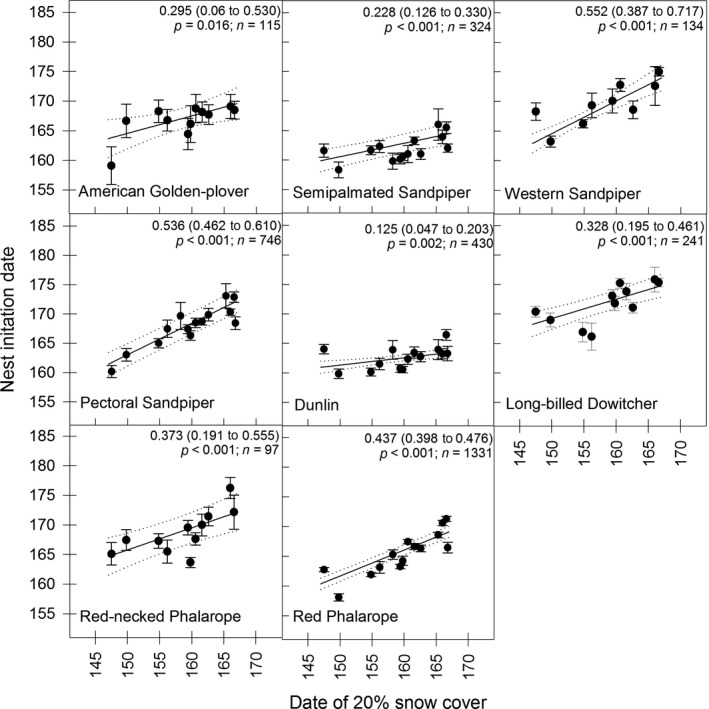
Observed (mean ± *SE*) and model predictions (line plot with 95% confidence intervals) for the advancement of nest initiation dates in response to earlier snowmelt for all shorebird species nesting near Utqiaġvik (formerly Barrow), Alaska, 2003–2016. Julian date 145 = 25 May (24 May in leap years). Parameter estimates (±*SE*) and *p*‐values for the effect of 20% snow cover, along with sample sizes are presented at the top of each panel for each species

We found that six of the eight shorebird species exhibited phenological advancement in laying dates (Figure 2), although no species appeared fully capable of advancing egg laying to keep pace with advancing snowmelt (i.e., all slopes significantly <1; Figure 3). Species that appeared most responsive to timing of snowmelt included Western Sandpiper (*Calidris mauri*), Pectoral Sandpiper (*Calidris melanotos*), and Red Phalarope, with slope parameters ranging from 0.44 to 0.55; all other species had slopes <0.4 (Figure 3). These trends remained consistent when comparing advancement rates, with Western Sandpiper, Pectoral Sandpiper, and Red Phalarope advancing egg laying by 0.34–0.98 days/year, while all other species advanced laying dates by <0.3 days/year (Figure 2). American Golden‐Plover (*Pluvialis dominica*) and Red‐necked Phalarope (*Phalaropus lobatus*) showed no significant advancement, although low sample sizes of these species may have reduced our ability to document advancement.

### 3.3 Role of migration pattern, timing of nesting, and settlement strategy on timing of egg laying

Little commonality existed in wintering location and migration strategies among species with similar responses to timing of snowmelt (see Table 1). However, we did find that species exhibiting an opportunistic settlement strategy (e.g., Pectoral Sandpiper and Red Phalarope) were more likely to respond to changing snowmelt conditions (i.e., larger slope parameters) as compared to species with a conservative settlement strategy (e.g., Dunlin and Semipalmated Sandpiper), while species exhibiting traits of both settlement strategies exhibited moderate response rates (see Table 1). This pattern, however, did not hold for Western Sandpiper which exhibited the highest response rate of all species, but exhibited traits of both settlement strategies (see Table 1). In addition, we found that species that historically nested the earliest (i.e., Dunlin and Semipalmated Sandpiper) also exhibited the lowest response rates as compared to later nesting species; however, as timing of nesting was correlated with settlement strategy, we were unable to differentiate which of these two life‐history characteristics was more important (see Table 1).

### 3.4 Individual phenotypic plasticity in timing of egg laying

Most individuals that returned to our study plots were resighted only once and typically in the following year (Dunlin: *n *= 142, 58% resighted once and 76% in the next year; Semipalmated Sandpipers: *n *= 80, 65% resighted once and 80% in the next year; Red Phalarope: *n *= 48, 81% resighted once and 58% in the next year; see also figure 3 in Saalfeld & Lanctot, 2015). Individuals showed a great deal of plasticity in when they nested, varying nest initiation dates by 21–27 days (mean = 6.0–6.6 days) among successive years, resulting in low repeatability of laying dates (Dunlin: *r *=* *−.040, *F*_141,237_ = 0.90, *p *= .761; Semipalmated Sandpiper: *r *= .079, *F*_79,128_ = 1.22, *p *= .155; and Red Phalarope: *r *= −0.144, *F*_47,59_ = 0.72, *p *= .877).

### 3.5 Role of experience and mate fidelity on timing of egg laying

We found little support to suggest that individuals learned from past site experience to better time nest initiation with snowmelt. Indeed, the difference between an individual's nest initiation date and date of 20% snow cover in the initial capture year was unrelated (*p *> .05) to the difference in the following year for all species except Semipalmated Sandpiper, which tended to always nest earlier in regard to timing of snow in the subsequent year (β = 0.328, 95% CI = 0.110–0.546, *F*_1,61_ = 8.67, *p *= .005).

We found no support to suggest repairing with a mate from a prior year increased the ability of birds to time nest initiation with snowmelt. In fact, there was no significant difference in the difference between an individual's nest initiation date and date of 20% snow cover between repaired and divorced pairs in both Dunlin (repaired: 0.36 ± 1.62, divorced: 4.95 ± 2.43, *F*_1,31_ = 2.10, *p *=* *.157, *n *= 33) and Semipalmated Sandpiper (repaired: −3.60 ± 1.40, divorced: −1.00 ± 1.51, *F*_1,9_ = 1.55, *p *= .245, *n *= 11), although there was a slight trend to initiate nests earlier with regard to snowmelt when paired with their old mate.

## 4 DISCUSSION

By the end of the century (i.e., 2100), climate change is predicted to advance the onset of summer by 8–11 days throughout the Arctic (Martin et al., 2009). However, at our study site in Utqiaġvik, Alaska, snowmelt is already occurring 11 days earlier than just 14 years ago (0.8 days/year). This rate is much faster than that estimated for the previous six decades in this region (0.13 days/year from 1940 to 2000; Stone, Dutton, Harris, & Longenecker, 2002). However, it should be noted that while we have one of the longest continuous datasets on shorebird nesting phenology, the time span of this analysis was still relatively short (14 years) in comparison with decades or century‐long climatic change predictions. In addition, our results may have been heavily influenced by conditions in the past few years (e.g., the last 2 years with extremely early snowmelt). Nonetheless, this drastic change in the start of summer is likely to have large consequences on the species inhabiting this region, especially migratory birds that may be especially vulnerable to phenological mismatch. As predicted, shorebirds in this study responded to earlier snowmelt by advancing nesting phenology (varying among species from 0.1–0.9 days earlier per year), although no species appeared capable of keeping pace with advancing snowmelt.

Previous studies have suggested that differences in adaptability among species may occur due to the variation in migration strategies (Both et al., 2010; Doxa et al., 2012; Jonzén et al., 2006; Saino et al., 2010; Visser et al., 2004). However, we found little commonality in migration patterns among species with similar adaptation abilities. One potential reason for this may be due to the sheer distances all species traveled between wintering and arctic‐breeding grounds, with even the shortest distance migrants traveling thousands of kilometers to winter in southern North America. Therefore, conditions experienced on wintering grounds, even for the shortest distance migrants, were likely unrelated to conditions in the Arctic, impeding the ability of any species to adjust departure times based on annual conditions. Migration route similarly did not appear to impact the ability of species to advance laying dates. Adjusting migration timing to breeding site conditions, however, is only possible if conditions experienced during migration are correlated with conditions on the breeding grounds, and species are able to alter migration speed (Ely, McCaffery, & Gill, in press). However, our assessments of the effects of migration patterns on nest initiation timing were quite coarse; more information is needed on specific migratory routes and stop‐over locations and durations used by species to assess how environmental factors at staging and breeding sites influence the ability of species to adapt to earlier summers.

Regardless of migration route, the date when species first arrive on the breeding grounds relative to the start of egg laying may constrain a species’ ability to advance their nesting phenology. Indeed, the two species (i.e., Dunlin and Semipalmated Sandpiper) that nested the earliest on our study plots also responded the least to timing of snowmelt. Although we do not have exact arrival dates of species investigated in this study, it seems likely that these species are already nesting as early as possible given current arrival dates. For example, it was estimated that, on average, Dunlin is currently nesting just 4–7 days after arrival (Doll, 2013). Therefore, shortening the period between arrival and egg laying may not be possible for these early nesting species.

In addition to timing of egg laying, settlement strategy may also influence a species’ ability to advance nesting phenology, with species exhibiting an opportunistic settlement strategy potentially more likely to respond to changing snowmelt conditions than species with a conservative strategy. This may be due to the ability of opportunistic species to travel across large portions of their breeding range while assessing breeding site conditions and ultimately making adjustments in timing that conservative species may be unable to achieve (Kempenaers & Valcu, 2017; Lanctot & Weatherhead, 1997; Lanctot et al., 2016). The highly variable conditions at Utqiaġvik (e.g., mean annual snowmelt ranged from 28 May to 16 June) may limit the ability of conservative species to use the past experiences to react to future conditions. Surprisingly, even the additional benefits from repairing with the same mate did not allow conservative species to adjust adequately, although repairing with the same mate did result in slightly earlier egg laying dates, although not significantly. Western Sandpipers, however, did not follow this general trend, with this species exhibiting the greatest response to timing of snowmelt despite having both conservative and opportunistic traits. Why this species did not conform to the general pattern is unclear but could be due to this species being on the fringe of its breeding range in Utqiaġvik with numbers increasing since 2008. Therefore, changes to the population occurring at the same time as changes in snowmelt conditions may have confounded our results. More work, however, is needed to tease apart these potentially confounding factors and to verify the qualitative analyses conducted here.

A comparison of our results to published literature on phenological advancement rates in shorebird species and sites across the Arctic revealed similar rates and explanatory patterns. Advancement rates were generally ≤0.5 days/year for most species, but with greater advancement in late‐laying and opportunistic species (Grabowski et al., 2013; Høye et al., 2007; Liebezeit et al., 2014; McKinnon et al., 2012). For example, at Prudhoe Bay, Alaska, phalaropes characterized as late‐laying opportunistic species, exhibited greater phenological advancement (0.8 days/year) than Pectoral Sandpiper (0.5 days/year), an early‐laying opportunistic species, and Semipalmated Sandpiper (0.4 days/year), an early‐laying conservative species (Liebezeit et al., 2014). Similar results were also found at Bylot Island, Canada (McKinnon et al., 2012), where White‐rumped Sandpiper (*Calidris fuscicollis*), a late‐laying opportunistic species, was the only species to significantly advance egg laying dates (0.1 days/year). Other species investigated at this site were early‐laying conservative species (i.e., American Golden‐Plover and Baird's Sandpiper [*Calidris bairdii*]). At other sites in Northeast Greenland and on Herschel Island in Canada, early‐laying conservative species such as Ruddy Turnstone (*Arenaria interpres*), Sanderling (*Calidris alba*), Dunlin, and Baird's Sandpiper advanced egg laying dates by 0.4–1.0 days/year; however, no late‐laying or opportunistic species were available at these sites for comparison (Grabowski et al., 2013; Høye et al., 2007); therefore, it is unknown if the patterns at these sites contradict or conform to our observed patterns. Overall, these comparisons illustrate that, in general, early‐laying conservative species were the least adaptable throughout their range; however, more work is needed to verify these qualitative patterns.

The current inability of many species to keep pace with earlier summers in the Arctic is likely to have profound impacts on these species. This is because delayed nesting (relative to snowmelt) decreases the probability of hatching during peak insect emergence (Saalfeld et al. unpublished data), ultimately resulting in reduced juvenile growth and survival rates (Schekkerman et al., 2003; Pearce‐Higgins & Yalden, 2004; McKinnon et al., 2012, 2013; Reneerkens et al., 2016; Senner et al., 2016; Saalfeld et al. unpublished data). Further, delayed nesting reduces the time available for adults to renest should their first nest fail (Gates et al., 2013), for adults to rebuild body reserves lost due to egg laying and incubation duties, and for adults and chicks to acquire sufficient reserves for earlier and successful southbound migration (Meltofte et al., 2007; Taylor, Lanctot, Powell, Kendall, & Nigro, 2011; Tulp & Schekkerman, 2008). Earlier and warmer summers, however, are just one way climate change is affecting arctic‐breeding shorebirds. Climate change is also changing or projected to change the extent, quality, and location of breeding locations (Wauchope et al., 2016). As a result, migratory birds may need to fly longer distances or switch migratory routes to reach suitable breeding habitats; both of which, along with continual threats to wintering and migratory stop‐over locations, may result in the inability of species to accurately time phenology on the breeding grounds. Therefore, continuing to understand species‐specific adaptability remains important for determining the potential long‐term impacts under future climate change scenarios.

## CONFLICT OF INTEREST

None declared.

## AUTHOR CONTRIBUTIONS

STS and RBL conceived, designed, and executed this study. STS performed statistical analyses and wrote the manuscript. RBL provided editorial advice.

## References

[ece33517-bib-0001] Barto, L. , Pratt, M. W. , & Sinnett, D. (2015). Fox control on the Barrow Steller's eider conservation planning area: 2015 report. United States Department of Agriculture Animal and Plant Health Inspection Service, Wildlife Services: Palmer, AK, USA.

[ece33517-bib-0002] Bolduc, E. , Casajus, N. , Legagneux, P. , McKinnon, L. , Gilchrist, H. G. , Leung, M. , … Bêty, J. (2013). Terrestrial arthropod abundance and phenology in the Canadian Arctic: Modelling resource availability for Arctic‐nesting insectivorous birds. Canadian Entomologist, 145, 155–170.

[ece33517-bib-0003] Both, C. , Bouwhuis, S. , Lessells, C. M. , & Visser, M. E. (2006). Climate change and population declines in a long‐distance migratory bird. Nature, 441, 81–83. 16672969 10.1038/nature04539

[ece33517-bib-0004] Both, C. , van Asch, M. , Bijlsma, R. G. , van den Burg, A. B. , & Visser, M. E. (2009). Climate change and unequal phenological changes across four trophic levels: Constraints or adaptations? Journal of Animal Ecology, 78, 73–83. 18771506 10.1111/j.1365-2656.2008.01458.x

[ece33517-bib-0005] Both, C. , Van Turnhout, C. A. M. , Bijlsma, R. G. , Siepel, H. , Van Strien, A. J. , & Foppen, R. P. B. (2010). Avian population consequences of climate change are most severe for long‐distance migrants in seasonal habitats. Proceedings of the Royal Society Biological Sciences Series B, 1685, 1259–1266. 10.1098/rspb.2009.1525 PMC2842804 20018784

[ece33517-bib-0006] Bronson, F. H. (1985). Mammalian reproduction: An ecological perspective. Biology of Reproduction, 32, 1–26. 3882162 10.1095/biolreprod32.1.1

[ece33517-bib-0007] Brook, R. W. , Leafloor, J. O. , Abraham, K. F. , & Douglas, D. C. (2015). Density dependence and phenological mismatch: Consequences for growth and survival of sub‐arctic nesting Canada Geese. Avian Conservation and Ecology, 10, 1 https://doi.org/10.5751/ACE-00708-100101

[ece33517-bib-0008] Brown, J. , Everett, K. R. , Webber, P. J. , MacLean, S. F. Jr , & Murray, D. F. (1980). The coastal tundra at Barrow In Brown J., Miller P. C., Tieszen L. L. & Bunnell F. L. (Eds.), An arctic ecosystem: The coastal tundra at Barrow, Alaska (pp. 1–29). Stroudsburg, PA, USA: Dowden, Hutchinson, and Ross.

[ece33517-bib-0009] Callaghan, T. V. , Bjorn, L. O. , Chernov, Y. I. , Chapin, T. , Christensen, T. R. , Huntley, B. , … Oechel, W. (2005). Arctic tundra and polar desert ecosystems In Symon C., Arris L. and Heal B. (Eds.), Arctic climate impact assessment (pp. 243–352). Cambridge: Cambridge University Press.

[ece33517-bib-0010] Crick, H. Q. P. , Dudley, C. , Glue, D. E. , & Thomson, D. L. (1997). UK birds are laying eggs earlier. Nature, 388, 526.

[ece33517-bib-0011] Danks, H. V. (1999). Life cycles in polar arthropods ‐ flexible or programmed? European Journal of Entomology, 96, 83–102.

[ece33517-bib-0012] Doiron, M. , Gauthier, G. , & Lévesque, E. (2015). Trophic mismatch and its effects on the growth of young in an Arctic herbivore. Global Change Biology, 21, 4364–4376. 26235037 10.1111/gcb.13057

[ece33517-bib-0013] Doll, A. C. (2013). Tracking spatiotemporal movements of Dunlin (Calidris alpina articola) migration through stable isotope analysis. MS Thesis, University of Colorado, Boulder, CO, USA.

[ece33517-bib-0014] Doxa, A. , Robert, A. , Crivelli, A. , Catsadorakis, G. , Naziridis, T. , Nikolaou, H. , … Theodorou, K. (2012). Shifts in breeding phenology as a response to population size and climatic change: A comparison between short‐ and long‐distance migrant species. The Auk: Ornithological Advances, 129, 753–762.

[ece33517-bib-0015] Durant, J. M. , Hjermann, D. Ø. , Ottersen, G. , & Stenseth, N. C. (2007). Climate and the match or mismatch between predator requirements and resources availability. Climate Research, 33, 271–283.

[ece33517-bib-0016] Ely, C. R. , McCaffery, B. J. , & Gill, R. E. (in press). Shorebirds adjust spring arrival schedules with variable environmental conditions; a 4‐decade assessment on the Yukon Kuskokwim Delta, Alaska. Western Birds.

[ece33517-bib-0017] Forchhammer, M. C. , Post, E. , & Stenseth, N. C. (1998). Breeding phenology and climate. Nature, 391, 29–30.9422504

[ece33517-bib-0018] Gaston, A. J. , Gilchrist, H. G. , Mallory, M. L. , & Smith, P. A. (2009). Changes in seasonal events, peak food availability, and consequent breeding adjustment in a marine bird: A case of progressive mismatching. Condor, 111, 111–119.

[ece33517-bib-0019] Gates, H. R. , Lanctot, R. B. , & Powell, A. N. (2013). High renesting rates in Arctic‐breeding Dunlin (*Calidris alpina*): A clutch‐removal experiment. The Auk: Ornithological Advances, 130, 372–380.

[ece33517-bib-0020] Gill, J. A. , Alves, J. A. , Sutherland, W. J. , Appleton, G. F. , Potts, P. M. , & Gunnarsson, T. G. (2014). Why is timing of bird migration advancing when individuals are not? Proceedings of the Royal Society Biological Sciences Series B, 281, 20132161 https://doi.org/20132110.20131098/rspb.20132013.20132161 10.1098/rspb.2013.2161 PMC3843828 24225454

[ece33517-bib-0021] Grabowski, M. M. , Doyle, F. I. , Reid, D. G. , Mossop, D. , & Talarico, D. (2013). Do Arctic‐nesting birds respond to earlier snowmelt? A multi‐species study in north Yukon, Canada. Polar Biology, 36, 1097–1105.

[ece33517-bib-0022] Green, G. H. , Greenwood, J. J. D. , & Lloyd, C. S. (1977). The influence of snow conditions on the date of breeding of wading birds in north‐east Greenland. Journal of Zoology, 183, 311–328.

[ece33517-bib-0023] Harrington, R. , Woiwod, I. , & Sparks, T. (1999). Climate change and trophic interactions. Trends in Ecology & Evolution, 14, 146–150. 10322520 10.1016/s0169-5347(99)01604-3

[ece33517-bib-0024] Helm, B. , Gwinner, E. , & Trost, L. (2005). Flexible seasonal timing and migratory behavior: Results from Stonechat breeding programs. Annals of the New York Academy of Sciences, 1046, 216–227. 16055855 10.1196/annals.1343.019

[ece33517-bib-0025] Hodgkins, R. (2014). The twenty‐first‐century arctic environment: Accelerating change in the atmospheric, oceanic and terrestrial spheres. The Geographical Journal, 180, 429–436.

[ece33517-bib-0026] Høye, T. T. , & Forchhammer, M. C. (2008). Phenology of high‐arctic arthropods: Effects of climate on spatial, seasonal, and inter‐annual variation. Advances in Ecological Research, 40, 299–324.

[ece33517-bib-0027] Høye, T. T. , Post, E. , Meltofte, H. , Schmidt, N. M. , & Forchhammer, M. C. (2007). Rapid advancement of spring in the high arctic. Current Biology, 17, 449–451. 10.1016/j.cub.2007.04.047 17580070

[ece33517-bib-0028] Jehl, J. R. Jr (1973). Breeding biology and systematic relationships of the stilt sandpiper. Wilson Bulletin, 85, 115–147.

[ece33517-bib-0029] Jönsson, P. E. (1987). Sexual size dimorphism and disassortative mating in the Dunlin *Calidris alpina schinzii* in southern Sweden. Ornis Scandinavica, 18, 257–264.

[ece33517-bib-0030] Jonzén, N. , Lindén, A. , Ergon, T. , Knudsen, E. , Vik, J. O. , Rubolini, D. , … Stenseth, N. C. (2006). Rapid advance of spring arrival dates in long‐distance migratory birds. Science, 312, 1959–1961. 16809542 10.1126/science.1126119

[ece33517-bib-0031] Karagicheva, J. , Rakhimberdiev, E. , Dekinga, A. , Brugge, M. , Koolhaas, A. , ten Horn, J. , & Piersma, T. (2016). Seasonal time keeping in a long‐distance migrating shorebird. Journal of Biological Rhythms, 31, 509–521. 27466352 10.1177/0748730416655929

[ece33517-bib-0032] Kempenaers, B. , & Valcu, M. (2017). Breeding site sampling across the Arctic by individual males of a polygynous shorebird. Nature, 541, 528–531. 28068667 10.1038/nature20813

[ece33517-bib-0033] Kerby, J. , & Post, E. (2013). Capital and income breeding traits differentiate trophic match–mismatch dynamics in large herbivores. Philosophical Transactions of the Royal Society of London B Biological Sciences, 368, 20120484 https://doi.org/20120410.20121098/rstb.20122012.20120484 23836789 10.1098/rstb.2012.0484 PMC3720056

[ece33517-bib-0034] Lanctot, R. B. , & Weatherhead, P. J. (1997). Ephemeral lekking behavior in the Buff‐breasted Sandpiper, *Tryngites subruficollis* . Behavioral Ecology, 8, 268–278.

[ece33517-bib-0035] Lanctot, R. B. , Yezerinac, S. , Aldabe, J. , Bosi de Almeida, J. , Castresana, G. , Brown, S. , … Fox, J. W. (2016). Light‐level geolocation reveals migration patterns of the Buff‐breasted Sandpiper. Wader Study, 123, 29–43.

[ece33517-bib-0036] Lessells, C. M. , & Boag, P. T. (1987). Unrepeatable repeatabilities: A common mistake. The Auk: Ornithological Advances, 104, 116–121.

[ece33517-bib-0037] Liebezeit, J. R. , Gurney, K. E. B. , Budde, M. , Zack, S. , & Ward, D. (2014). Phenological advancement in arctic bird species: Relative importance of snow melt and ecological factors. Polar Biology, 37, 1309 https://doi.org/1310.1007/s00300-00014-01522-x

[ece33517-bib-0038] Liebezeit, J. R. , Smith, P. A. , Lanctot, R. B. , Schekkerman, H. , Tulp, I. , Kendall, S. J. , … Zack, S. W. (2007). Assessing the development of shorebird eggs using the flotation method: Species‐specific and generalized regression models. Condor, 109, 32–47.

[ece33517-bib-0039] Martin, P. D. , Jenkins, J. L. , Adams, F. J. , Jorgenson, M. T. , Matz, A. C. , Payer, D. C. , … Zelenak, J. R. (2009). Wildlife response to environmental Arctic change: predicting future habitats of Arctic Alaska. Report of the Wildlife Response to Environmental Arctic Change (WildREACH): Predicting Future Habitats of Arctic Alaska Workshop, 17‐18 November 2008. Fairbanks, AK, USA: US Fish and Wildlife Service.

[ece33517-bib-0040] McKinnon, L. , Nol, E. , & Juillet, C. (2013). Arctic‐nesting birds find physiological relief in the face of trophic constraints. Scientific Reports, 3, 1–6. 10.1038/srep01816 PMC3648796 23657421

[ece33517-bib-0041] McKinnon, L. , Picotin, M. , Bolduc, E. , Juillet, C. , & Bêty, J. (2012). Timing of breeding, peak food availability, and effects of mismatch on chick growth in birds nesting in the High Arctic. Canadian Journal of Zoology, 90, 961–971.

[ece33517-bib-0042] Meltofte, H. (1985). Populations and breeding schedules of waders, Charadrii, in high arctic Greenland. Meddelelser om Grønland. BioScience, 16, 1–43.

[ece33517-bib-0043] Meltofte, H. , Høye, T. T. , Schmidt, N. M. , & Forchhammer, M. C. (2007). Differences in food abundance cause inter‐annual variation in the breeding phenology of High Arctic waders. Polar Biology, 30, 601–606.

[ece33517-bib-0044] Miller, E. H. (1983). Habitat and breeding cycle of the Least Sandpiper (*Calidris minutilla*) on Sable Island, Nova Scotia. Canadian Journal of Zoology, 61, 2880–2898.

[ece33517-bib-0045] Møller, A. P. , Rubolini, D. , & Lehikoinen, E. (2008). Populations of migratory bird species that did not show a phenological response to climate change are declining. Proceedings of the National Academy of Sciences, 105, 16195–16200. 10.1073/pnas.0803825105 PMC2571031 18849475

[ece33517-bib-0046] Naves, L. C. , Lanctot, R. B. , Taylor, A. R. , & Coutsoubos, N. P. (2008). How often do Arctic shorebirds lay replacement clutches? Wader Study Group Bulletin, 115, 2–9.

[ece33517-bib-0047] Parmesan, C. , & Yohe, G. (2003). A globally coherent fingerprint of climate change impacts across natural systems. Nature, 421, 37–42. 12511946 10.1038/nature01286

[ece33517-bib-0048] Pearce‐Higgins, J. W. , & Yalden, D. W. (2004). Habitat selection, diet, arthropod availability and growth of a moorland wader: The ecology of European Golden Plover *Pluvialis apricaria* chicks. Ibis, 146, 335–346.

[ece33517-bib-0049] Pearce‐Higgins, J. W. , Yalden, D. W. , & Whittingham, M. J. (2005). Warmer springs advance the breeding phenology of golden plovers *Pluvialis apricaria* and their prey (Tipulidae). Oecologia, 143, 470–476. 15685442 10.1007/s00442-004-1820-z

[ece33517-bib-0050] Piersma, T. , Brugge, M. , Spaans, B. , & Battley, P. F. (2008). Endogenous circannual rhythmicity in body mass, molt, and plumage of great knots (*Calidris tenuirostris*). The Auk: Ornithological Advances, 125, 140–148.

[ece33517-bib-0051] Poole, A. , editor (2005). The birds of North America online. Ithaca, NY, USA: Cornell Laboratory of Ornithology Retrieved from http://bna.birds.cornell.edu/BNA/.

[ece33517-bib-0052] Post, E. , Forchhammer, M. C. , Stenseth, N. C. , & Callaghan, T. V. (2001). The timing of life‐history events in a changing climate. Proceedings of the Royal Society Biological Sciences Series B, 268, 15–23. 10.1098/rspb.2000.1324 PMC1087595 12123293

[ece33517-bib-0053] Priklonsky, S. G. (1960). Application of small automatic bows for catching birds. Zoologicheskii Zhurnal, 39, 623–624.

[ece33517-bib-0054] Reneerkens, J. , Schmidt, N. M. , Gilg, O. , Hansen, J. , Hansen, L. H. , Moreau, J. , & Piersma, T. (2016). Effects of food abundance and early clutch predation on reproductive timing in a high Arctic shorebird exposed to advancements in arthropod abundance. Ecology and Evolution, 6, 7375–7386. https://doi.org/10.1002/ece3.2361 28725405 10.1002/ece3.2361 PMC5513252

[ece33517-bib-0055] Saalfeld, S. T. , & Lanctot, R. B. (2015). Conservative and opportunistic settlement strategies in Arctic‐breeding shorebirds. The Auk: Ornithological Advances, 132, 212–234.

[ece33517-bib-0056] Saino, N. , Ambrosini, R. , Rubolini, D. , von Hardenberg, J. , Provenzale, A. , Hüppop, K. , … Sokolov, L. (2010). Climate warming, ecological mismatch at arrival and population decline in migratory birds. Proceedings of the Royal Society Biological Sciences Series B, 278, 835–842. 10.1098/rspb.2010.1778 PMC3049050 20861045

[ece33517-bib-0057] Sandercock, B. K. , Lank, D. B. , & Cooke, F. (1999). Seasonal declines in the fecundity of Arctic‐breeding sandpipers: Different tactics in two species with an invariant clutch size. Journal of Avian Biology, 30, 460–468.

[ece33517-bib-0058] Schekkerman, H. , Tulp, I. , Piersma, T. , & Visser, G. H. (2003). Mechanisms promoting higher growth rate in arctic than in temperate shorebirds. Oecologia, 134, 332–342. 12647140 10.1007/s00442-002-1124-0

[ece33517-bib-0059] Senner, N. R. , Stager, M. , & Sandercock, B. K. (2016). Ecological mismatches are moderated by local conditions for two populations of a long‐distance migratory bird. Oikos, 126, 61–72.

[ece33517-bib-0060] Serreze, M. C. , & Francis, J. A. (2006). The Arctic amplification debate. Climate Change, 76, 241–264.

[ece33517-bib-0061] Smith, P. A. , Gilchrist, H. G. , Forbes, M. R. , Martin, J.‐L. , & Allard, K. (2010). Inter‐annual variation in the breeding chronology of arctic shorebirds: Effects of weather, snow melt and predators. Journal of Avian Biology, 41, 292–304.

[ece33517-bib-0062] Soikkeli, M. (1967). Breeding cycle and population dynamics in the Dunlin (*Calidris alpina*). Annales Zoologici Fennici, 4, 158–198.

[ece33517-bib-0063] Stenseth, N. C. , Mysterud, A. , Ottersen, G. , Hurrell, J. W. , Chan, K.‐S. , & Lima, M. (2002). Ecological effects of climate fluctuations. Science, 297, 1292–1296. 12193777 10.1126/science.1071281

[ece33517-bib-0064] Stone, R. S. , Dutton, E. G. , Harris, J. M. , & Longenecker, D. (2002). Earlier spring snowmelt in northern Alaska as an indicator of climate change. Journal of Geophysical Research, 107, 4089.

[ece33517-bib-0065] Taylor, A. R. , Lanctot, R. B. , Powell, A. N. , Kendall, S. J. , & Nigro, D. A. (2011). Residence time and movements of postbreeding shorebirds on the northern coast of Alaska. Condor, 113, 779–794.

[ece33517-bib-0066] Thackeray, S. J. , Henrys, P. A. , Hemming, D. , Bell, J. R. , Botham, M. S. , Burthe, S. , … Wanless, S. (2016). Phenological sensitivity to climate across taxa and trophic levels. Nature, 535, 241–245. 27362222 10.1038/nature18608

[ece33517-bib-0067] Tulp, I. , & Schekkerman, H. (2008). Has prey availability for Arctic birds advanced with climate change? Hindcasting the abundance of tundra arthropods using weather and seasonal variation. Arctic, 61, 48–60.

[ece33517-bib-0068] Visser, M. E. (2008). Keeping up with a warming world; assessing the rate of adaptation to climate change. Proceedings of the Royal Society Biological Sciences Series B, 275, 649–659. 10.1098/rspb.2007.0997 PMC2409451 18211875

[ece33517-bib-0069] Visser, M. E. , Both, C. , & Lambrechts, M. M. (2004). Global climate change leads to mistimed avian reproduction. Advances in Ecological Research, 35, 89–110.

[ece33517-bib-0070] Visser, M. E. , Holleman, L. J. M. , & Gienapp, P. (2006). Shifts in caterpillar biomass phenology due to climate change and its impact on the breeding biology of an insectivorous bird. Oecologia, 147, 164–172. 16328547 10.1007/s00442-005-0299-6

[ece33517-bib-0071] Visser, M. E. , van Noordwijk, A. J. , Tinbergen, J. M. , & Lessells, C. M. (1998). Warmer springs lead to mistimed reproduction in Great Tits (*Parus major*). Proceedings of the Royal Society Biological Sciences Series B, 265, 1867–1870.

[ece33517-bib-0072] Walther, G.‐R. , Post, E. , Convey, P. , Menzel, A. , Parmesan, C. , Beebee, T. J. C. , … Bairlein, F. (2002). Ecological responses to recent climate change. Nature, 416, 389–395. 11919621 10.1038/416389a

[ece33517-bib-0073] Wauchope, H. S. , Shaw, J. D. , Varpe, Ø. , Lappo, E. G. , Boertmann, D. , Lanctot, R. B. , & Fuller, R. A. (2016). Rapid climate‐driven loss of breeding habitat for Arctic migratory birds. Global Change Biology, 23, 1085–1094. 27362976 10.1111/gcb.13404

[ece33517-bib-0074] Whelan, S. , Strickland, D. , Morand‐Ferron, J. , & Norris, D. R. (2016). Male experience buffers female laying date plasticity in a winter‐breeding, food‐storing passerine. Animal Behaviour, 121, 61–70.

